# A Genome-Wide SNP Scan Reveals Novel Loci for Egg Production and Quality Traits in White Leghorn and Brown-Egg Dwarf Layers

**DOI:** 10.1371/journal.pone.0028600

**Published:** 2011-12-08

**Authors:** Wenbo Liu, Dongfeng Li, Jianfeng Liu, Sirui Chen, Lujiang Qu, Jiangxia Zheng, Guiyun Xu, Ning Yang

**Affiliations:** National Engineering Laboratory for Animal Breeding and MOA Key Laboratory of Animal Genetics and Breeding, College of Animal Science and Technology, China Agricultural University, Beijing, China; University of Western Cape, South Africa

## Abstract

Availability of the complete genome sequence as well as high-density SNP genotyping platforms allows genome-wide association studies (GWAS) in chickens. A high-density SNP array containing 57,636 markers was employed herein to identify associated variants underlying egg production and quality traits within two lines of chickens, i.e., White Leghorn and brown-egg dwarf layers. For each individual, age at first egg (AFE), first egg weight (FEW), and number of eggs (EN) from 21 to 56 weeks of age were recorded, and egg quality traits including egg weight (EW), eggshell weight (ESW), yolk weight (YW), eggshell thickness (EST), eggshell strength (ESS), albumen height(AH) and Haugh unit(HU) were measured at 40 and 60 weeks of age. A total of 385 White Leghorn females and 361 brown-egg dwarf dams were selected to be genotyped. The genome-wide scan revealed 8 SNPs showing genome-wise significant (*P*<1.51E-06, Bonferroni correction) association with egg production and quality traits under the Fisher's combined probability method. Some significant SNPs are located in known genes including *GRB14* and *GALNT1* that can impact development and function of ovary, but more are located in genes with unclear functions in layers, and need to be studied further. Many chromosome-wise significant SNPs were also detected in this study and some of them are located in previously reported QTL regions. Most of loci detected in this study are novel and the follow-up replication studies may be needed to further confirm the functional significance for these newly identified SNPs.

## Introduction

Modern egg layers have been selected for egg production and quality for decades. A breeding program for egg-type chickens has to face two major problems, the measurement of phenotypic data for individual hens and the efficient selection of cockerels which do not have records on laying performance and egg quality. Although great success has been achieved in layer breeding over the past decades, it is becoming more and more difficult with traditional breeding programs to make improvement in egg production and quality traits. With advances in technologies of molecular genetics and availability of DNA markers, identifying QTL controlling egg production and quality traits for application in marker-assisted selection has been progressing rapidly [1], [2]. Through applying linkage analysis in reference mapping populations by using microsatellites and SNPs as markers, thousands of QTL for exterior, health, physiology and production traits were detected in chickens [3]. Out of them, 66 QTL were associated with 7 types of egg production traits including egg production rate, age at first egg, the number of eggs and so on, and 223 QTL were associated with 38 types of egg quality traits such as egg shell thickness, egg shell strength and yolk weight (data cited from Chicken QTLdb, http://www.animalgenome.org/cgi-bin/QTLdb/GG/index). In addition, through making association test based on direct genotypic effects of markers within or near candidate genes, several polymorphisms were revealed relevant to egg production and quality traits [4], [5], [6].

Although many studies have successfully identified lots of QTL and a few causative genes, application of these results in commercial lines is still infeasible due to the precision of mapping. Identification of numerous single nucleotide polymorphisms in animal genome [7], [8], advances in high-throughput genotyping methods [9], and progresses in developing computational methods for analyzing high-density SNP data [10] have made possible using genomic information in livestock breeding. The successes of genome-wide association studies (GWAS) for detection of loci affecting milk production, fertility and growth traits in cattle [11], [12], [13] has spurred interest in the use of high-density SNP genotyping platform for the identification of sequence variations influencing egg production and quality traits in chickens. A 60 K SNP Illumina iSelect chicken array developed by the USDA Chicken GWMAS Consortium is a new and powerful platform for polymorphism detection in the whole genome of the chicken. In this study, we performed a GWA study to discover genomic regions explaining variations in egg production and quality traits of White Leghorn and brown-egg dwarf layers developed at China Agricultural University [6], [14], [15] by using the 60K SNP Illumina chicken array.

## Materials and Methods

The blood samples were collected from brachial veins of chickens by standard venipuncture along with the regular quarantine inspection of the experimental station of China Agricultural University, and the whole procedure was carried out in strict accordance with the protocol approved by the Animal Welfare Committee of China Agricultural University (Permit Number: XK622).

### Animals and Data Collection

Two experimental lines of egg-type chickens, White Leghorn (WL) and dwarf brown egg layer (DW), have been selected for egg production for over 10 years in the experimental station of China Agricultural University. These two lines were employed as two different experimental populations in the study. For each line, 600 hens from 40 families were kept in individual cages and their egg productions were recorded daily from 21 to 56 weeks of age. Production traits including total number of eggs from 21 to 56 wk of age (EN), age at first egg(AFE), first egg weight (FEW) were summarized. Individual data for egg quality traits including egg weight (EW), eggshell weight (ESW), Yolk weight (YW), eggshell thickness (EST), eggshell strength (ESS), albumen height(AH) and Haugh unit(HU) were measured with conventional methods. Eggs were collected in 3 consecutive days when hens were 40 weeks and 60 weeks old. The average for 3 d was taken as phenotypic value of each trait for every hen. As egg production decreased with age, some hens laid only one egg or even none within the 3 test days at 40 or 60 wks, and therefore no egg quality data were available for those layers at the respective age. All phenotypic values of traits in genotyped individuals were tested for normality, and some abnormal values extremely deviating from normal distribution were deleted. Box-Cox transformation was made for traits apart from normal distribution before conducting association tests.

### Genotyping and Quality Control

Genomic DNA was isolated from blood sample by using standard phenol-chloroform extraction. Within family, two full-sib individuals with the same dam ID were randomly selected for SNP genotyping. In total, 385 White Leghorn females and 361 dwarf dams were genotyped for 57,636 markers by using 60K SNP Illumina iSelect chicken array. These markers cover twenty-nine autosomes including GGA1 to 28 and GGA 32, two linkage groups containing E22C19W28_E50C23 and E64, and two sex chromosomes (Table 1). The genotyping work was done by DNA LandMarks Inc., Quebec, Canada. Fourteen White Leghorn and 7 dwarf hens with an average SNP call rate <90% were excluded in the further analysis. We conducted quality control of SNP in two lines separately, and markers were selected based on three conditions: call rates were higher than 90%, minor allele frequencies were greater than 1%, and p-values for Hardy-Weinberg equilibrium tests were also greater than 1.00E-06. Finally, 37,518 SNP markers in WL and 43,991 SNP markers in DW remained after filtering (Table 1). In addition, as markers from GGA32, E64 and W are few in the 60K SNP array and most of them were discarded after quality control (Table 1), so all markers in these genomic regions were not included in further analysis.

**Table 1:** Distributions of SNPs in 60k SNP Illumina iSelect chicken array and their conditions after quality control.

		No.SNP remained after quality control		1Average distance (kb)	
GGA	No. SNP in chip	2WL	3DW	2WL	3DW
1	9059	6086	7163	33	28
2	6958	4324	5387	36	29
3	5171	3476	4072	33	28
4	4256	2907	3418	32	28
5	2766	1872	2171	33	29
6	2219	1549	1710	24	22
7	2280	1442	1742	26	22
8	1813	1268	1341	24	23
9	1504	1036	1182	25	22
10	1682	1078	1275	21	18
11	1647	929	1192	24	18
12	1671	1111	1280	18	16
13	1492	1043	1180	18	16
14	1284	859	1023	18	15
15	1337	855	1028	15	13
16	31	18	22	24	20
17	1104	730	891	15	13
18	1160	742	837	15	13
19	1053	709	792	14	13
20	1991	1233	1375	11	10
21	970	666	725	11	10
22	476	231	293	17	13
23	794	519	592	12	10
24	937	645	736	10	9
25	241	128	164	16	12
26	852	586	660	9	8
27	665	420	497	11	10
28	793	487	563	9	8
32	1	0	0	–	–
4E22	164	93	103	–	–
4E64	8	2	4	–	–
Z	3195	7	10	10714	7500
W	7	0	0	–	–
50	1211	467	563	–	–
Total	57636	37518	43991	–	–

1 Conditions after quality control.

2 White Leghorn.

3 Dwarf brown egg layer.

4 Linkage group.

5 These SNPs are not assigned to any chromosomes.

### Statistical Methods for Association Study

Association tests were performed based on generalized least squares (GLS) testing to account for sib correlation by using EPISNP computer package [16], which is applicable to all bi-allelic loci of diploid species, so Z chromosome loci in the female individual of chicken cannot be analyzed. Moreover, GLS test can also adjust phenotypic observation of individuals for family structure before significance test for each SNP. According to the manual of EPISNP, the statistical tests followed a two-step least square analysis. For the first step, phenotypic values were corrected for fixed non-genetic effects, but in the present study all experimental chickens came from one generation with the same gender and they were raised in the same house under same environments, so there was no known fixed non-genetic effect impacting the results of association tests. In the second step, single-locus tests using the phenotypic values were conducted. The statistical model is:
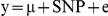
where y = phenotypic value corrected by fixed non-genetic effect, μ = common mean, SNP = the single-locus SNP genotypic effect, and e = random residual. The single-locus SNP genotypic value was partitioned into additive and dominance effect. The extended Kempthorne model was applied for testing additive and dominant effects of each SNP in EPISNP [17]. In this model, a t-test was used to test the significance of additive and dominance using the following formula:
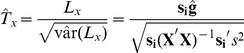
where *L_x_* = contrast to estimate the genetic effect, **s_i_** = a function of marginal and conditional allelic and genotypic frequencies for estimating additive or dominance effect, 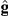 = the least squares estimates of the SNP genotypic effects, 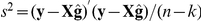 = estimated residual variance, **y** = vector of phenotypic values, **X** = the design matrix, *n* = number of observations, *k* = rank of **X** [16], [17]. White Leghorn and dwarf layers were analyzed independently. Results obtained from EPISNP were checked by using the linear model in PLINK [18].

As White Leghorn and dwarf layers are different in their genetic background and phenotypic values (Table 2), it is not suitable to incorporate data from these two populations in simultaneous association tests. A method for combined *P* values originally proposed by Fisher from independent tests of significance is adopted herein to address the heterogeneity of raw data. This strategy has also been successfully applied to GWAS and expression arrays elsewhere [19], [20], [21]. In this study, for a SNP marker, let *P*_wl_ and *P*_dw_ be the *P* value for significance of SNP effect (additive or dominance) obtained from association tests in White Leghorn and dwarf layer population separately, and s = −2ln(*P*_wl_+*P*_dw_). Then under H0, *P*_wl(dw)_∼unif (0,1). Hence s∼χ^2^(df = 4). H0 can be rejected at α level of significance if s>χ^2^(1−α,df = 4). Bonferroni method was adopted to adjust for multiple testing from the number of SNP markers detected. A significant SNP was declared if its combined *P* value<0.05/N, here N is the number of SNP markers tested in combined analyses.

**Table 2:** Traits analyzed in White Leghorn and dwarf layers with phenotypic mean, standard deviation, and number of chickens with records.

	White Leghorn			Dwarf layer		
Trait	Mean	SD	n	Mean	SD	n
Number of egg at 21–56 wk (EN)^1^	196.0	26.0	385	194.2	23.5	361
Age at first egg (AFE, day)^1^	152.1	9.2	385	148.7	22.8	361
First egg weight (FEW, g)^1^	39.80	5.20	385	38.58	7.62	361
Egg weight at 40 wk (EW40, g)	55.46	3.80	385	53.78	3.96	361
Egg weight at 60 wk (EW60, g)	59.90	4.06	275	59.36	5.07	308
Egg shell weight at 40 wk (ESW40,g)	7.74	1.03	385	7.20	0.84	361
Egg shell weight at 60 wk (ESW60,g)^2^	6.96	0.66	280	6.53	0.63	307
Yolk weigh at 40 wk (YW40, g)	15.51	1.05	385	14.84	1.23	361
Yolk weigh at 60 wk (YW60, g)	17.29	1.17	275	16.99	1.68	308
Egg shell strength at 40 wk (ESS40, Kg/cm2)	3.046	0.647	385	2.976	0.619	361
Egg shell strength at 60 wk (ESS60, Kg/cm2)^3^	2.888	0.612	281	2.719	0.649	320
Egg shell thickness at 40 wk (EST40, mm)^2^	0.316	0.024	385	0.301	0.024	361
Egg shell thickness at 60 wk (EST60, mm)	0.329	0.028	279	0.306	0.029	313
Albumen height at 40 wk (AH40, mm)	6.2	1.0	385	6.6	1.0	361
Albumen height at 60 wk (AH60, mm)	5.8	0.9	277	6.4	1.0	316
Haugh unit at 40 wk (HU40)	79.6	8.4	385	83.2	7.7	361
Haugh unit at 60 wk (HU60)^1^	75.3	6.8	273	79.2	7.2	316

1 Phenotypic values of traits in the both two strains are not within the normal distribution.

2 Phenotypic values of EST40 and ESW60 in WL are not within the normal distribution.

3 Phenotypic values of ESS60 in DW are not within the normal distribution.

## Results

Descriptive statistics of phenotypic measurements of egg quality and production traits in White Leghorn and dwarf layers used for GWAS were given in Table 2. All non-normal phenotypic data are within normal ranges after the Box-Cox transforming (Table 3).

**Table 3:** Phenotypic mean, standard deviation and status of normalization for non-normal traits after the transformation.

	White Leghorn			Dwarf layer			
Trait	Mean	SD	n	Mean	SD	n	Status of normalization
EN	7.66E05	2.42E05	385	7.37E05	2.25E05	361	Yes
AFE	4.38E-05	5.12E-06	385	6.66E-01	3.23E-05	361	Yes
FEW	6.29	0.39	385	3.66	0.12	361	Yes
HU60	7478.14	1465.22	273	1.71E05	4.04E04	316	Yes
EST40^1^	7.18E05	1.29E05	385	–	–	–	Yes
ESW60^1^	11.63	1.69	280	–	–	–	Yes
ESS60^2^	–	–	–	1.276	0.394	320	Yes

1 EST40 and ESW60 have been transformed only for the White Leghorns.

2 ESS60 has been transformed only for the dwarf layers.

**Figure 1:**
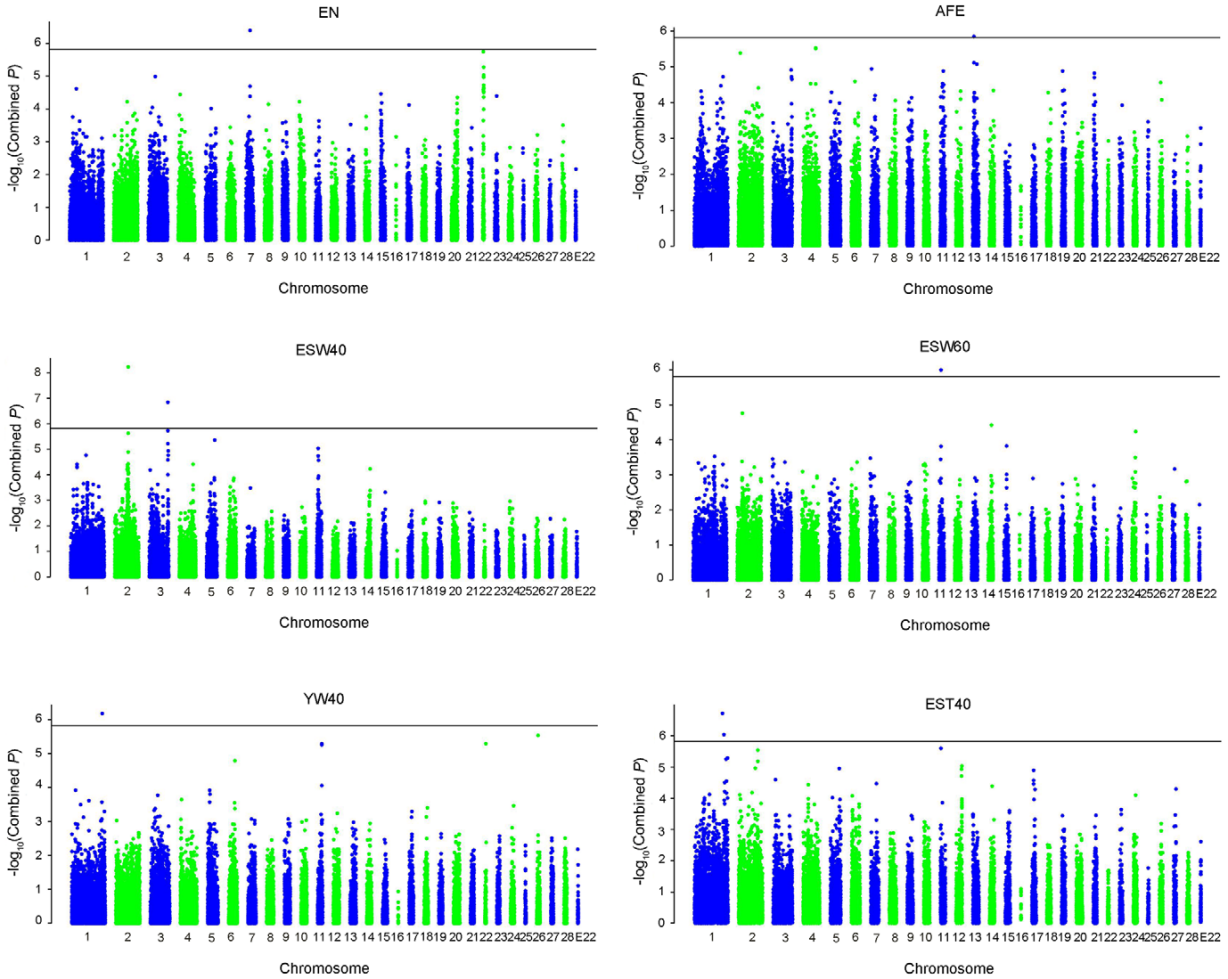
Genome-wide scan for egg production and quality traits: −log10 of the combined P value analysis for association with SNPs. Chromosome 1–28 and linkage group E22 are shown in alternating colors for clarity. The horizontal lines indicate the genome-wise significance threshold: −log10 (1.51E-06).

Association tests were performed separately in White Leghorn and dwarf layer populations, and all results obtained from EPISNP were re-analyzed with PLINK. As PLINK also uses least squares regression analysis for quantitative traits, the results from PLINK were almost the same as those from EPISNP. Subsequently, *P* values from the two independent analyses for WL and DW were combined under the Fisher's method by using 33,068 markers shared in the two populations. Taking 1.51E-06 (0.05/N, N = 33,068) as the genome-wise significance level with Bonferroni correction, it was revealed that 8 additive SNP effects showed significant association with egg production and quality traits including AFE, EN, ESW40, ESW60, EST40 and YW40. No dominance effects reached genome-wise significance. The profiles of *P* values, in terms of −log (*P*), of all tested SNPs after combining for different traits are shown in Figure 1. Furthermore, considering that Bonferroni correction is overly conservative and may lead to high proportion of negative false as marker density increase [22], we also tested association at chromosome-wide significance level, and the threshold ranged from 9.12E-06 on GGA1 to 6.67E-04 on E22 linkage group (Table S1). A total of 95 additive SNP effects exceeded the chromosome-wide significance threshold (Table S2). A few dominance effects reached chromosome-wise significance but were less significant (data not shown). Application of the new chicken SNP array allowed genotyping at a higher marker density than most previous studies, hence some previously undetected loci for egg quality and production traits were found in the present study. The details of all genome-wise significant SNPs, including their positions in the genome, raw *P* value in each population, combined *P* values, and candidate genes are summarized in Table 4 and further described as follows.

**Table 4:** Genome-wise significant (P<1.51E-06, Bonferroni correction) SNPs for egg production and quality traits.

SNP	Associated Traits	GGA	Position (bp)	1Pwl	1Pdw	2Combined P	Candidate gene
GGaluGA315030	EN	7	21676854	6.17E-01	3.45E-08	3.97E-07	GRB14
GGaluGA092322	AFE	13	4796267	6.85E-06	1.19E-02	1.42E-06	ODZ2
rs13636444	ESW40	2	86114050	6.92E-10	3.66E-01	5.85E-09	GALNT1
rs14411624	ESW40	3	110095288	1.40E-07	5.09E-02	1.41E-07	BLK
rs14022717	ESW60	11	9596922	2.24E-07	2.49E-01	8.62E-07	ZNF536
GGaluGA059301	YW40	1	184632695	3.31E-02	1.1E-06	2.56E-08	ATM
rs13968878	EST40	1	171224927	6.91E-08	1.41E-01	2.81E-08	ENOX1
rs13978498	EST40	1	179350984	1.74E-02	2.97E-06	9.22 E-07	LOC418918

1 Results of independent association test in White Leghorn and dwarf layer populations.

2 Combined P value follow the Fisher's method.

### Age at First Egg (AFE) and Number of Eggs (EN)

A SNP on GGA13 was found to be significantly associated with AFE (combined *P* = 1.42E-06), and it was located in the intron2 of odd Oz/ten-m homolog 2 gene (*ODZ2*). For EN, one SNP at 21.67 Mb on GGA7 with combined *P* value of 3.97E-07 showed association, and it was located in the intron12 of growth factor receptor-bound protein 14 gene (*GRB14*).

### Egg Shell Weight (ESW) and Yolk Weight (YW)

As egg weight has a great impact on eggshell and yolk weight, it was taken as a covariant in the association test for ESW and YW. Two significant SNPs were detected by combined analysis for ESW40, one on GGA2 (combined *P* = 5.85E-09) and the other on GGA3 (combined *P* = 1.41E-07). The significant SNP on GGA2 is located in the intron2 of UDP-N-acetyl-alpha-D-galactosamine:polypeptide N-acetylgalactosaminyltransferase 1 gene (*GALNT1*). The significant SNP on GGA3 is at site of 100.09 Mb and 21,753 bps away from the nearest known gene *BLK*. An association signal was also detected in analysis of ESW60, which is at about 9.59 Mb on GGA11 and in the intron3 of zinc finger protein 536 gene (*ZNF536*). For YW40, only one SNP at 184.63 Mb on GGA1 reached the genome-wise significant level, which is located in ataxia telangiectasia mutated gene (*ATM*).

### Egg Shell Thickness (EST)

Two SNPs located within an 8.12 Mb segment (between 171.22 Mb to 179.35 Mb) on GGA1 were found to be significantly associated with EST40 by using combined *P* method. One of them (combined *P* = 9.21E-07) is located in a predicted gene *LOC418918*, and the other one (combined *P* = 1.89E-07) is about 22,428 bps away from a known gene *ENOX1*.

### Chromosome-wise Significant SNPs

For other phenotypes including AH and HU, the combined *P* values of association tests did not reach the genome-wise significant level, but some SNP-trait combinations showed suggestive evidence with chromosome-wise significant level (Table S1), especially with SNPs in QTL regions identified in previous studies, which can provide valuable references for subsequent researches.

## Discussion

For domestic animals, the genome-wide association study is becoming a powerful approach for genetic dissection of trait loci along with the completion of genome sequencing and development of high density SNP array. Recently, GWAS has already been applied in the cattle and revealed several loci impacting economically important traits [11], [12], [13]. Following successful application of GWAS in cattle, we conducted a GWAS in White Leghorn and dwarf layer populations and provided strong evidences for association of SNPs with 7 traits of egg production and quality. A remarkable aspect of our study is that most SNPs found at genome-wide significance level are within the known genes, indicating that there are disequilibrium between the marker SNP and the causative variation within or near genes, though the characteristics and functions of these genes have not been studied in depth. Identifications of these loci may provide new insights into the genetic basics of egg production and quality traits. Another notable aspect of the present study is that most of the significant SNP were additive with little dominance detected. The reason for this result may be that the dominance variation is less in purebred laying hens than in crossbred ones [23]. Furthermore, for some egg production traits such as EN and EW, dominant variance just presents a small ratio of total phenotypic variance [24]. Similarly little dominance effects with genome-wise significance were detected in our study.

Number of eggs and age at first egg are two important production traits in layers, and producing hens with earlier sexual maturity and higher rate of lay has always been the goal of egg-type chicken breeding. As these reproductive traits are sex-limited and have low to moderate heritability, they would greatly benefit from marker-assisted selection, where the selection can be directed towards actual genetic variation. In this study, a most significant SNP associated with egg number was found to be located in the intron12 of *GRB14* gene that encodes a growth factor receptor-binding protein. In human and mammals, *GRB14* mRNA was found to be expressed at high level in the ovary, liver, kidney, skeletal muscle and so on [25], [26]. It interacts with insulin receptors (IR) and insulin-like growth-factor receptors (IGFR), and may play an inhibiting role in tyrosine kinase receptor (Tkr) signaling pathways [27], [28]. IGF and IGFR were reported to regulate ovarian functions and follicular developments in chickens [29], [30]. Although the function of *GRB14* in chicken is undefined, it may combine with the IGF system to influence egg production in layers.

Age at first egg is an indicator of sexual maturity, and can be impacted by several factors including nutrition, photoperiod and genetics. In the present study, a SNP in the intron2 of *ODZ2* gene was revealed to be associated significantly with Age at first egg. *ODZ2*, also known as *Teneurin-2*, encodes a neuronal cell surface protein and plays an important role in development of nervous system [31]. It was found that *Teneurin* are expressed prominently in developing chicken brain, and especially in the visual system including retina and optic tectum [32]. The current study provides the first report that *Teneurin-2* may have effect on the sexual maturity of chickens. A recent study found that expressions of genes in the nervous system can influence the age when chickens lay their first egg [33]. Furthermore, some previous studies revealed that light intensity can influence layers' age at first egg and longer light periods can lead to earlier sexual maturity [34], [35]. As stimulations of lights play roles mainly through the visual and nervous system, genes related to these systems may impact sexual maturity in chickens.

In addition to egg production, egg quality is another major selection criterion in poultry breeding, especially eggshell quality. Good eggshell quality is not only important for reproductive performance but also for human consumption. Eggshell weight, eggshell strength, and eggshell thickness are important indications of eggshell quality. We identified several significant SNPs influencing eggshell weight at different age. One significant SNP associated with ESW40 is in the intron2 of *GALNT1* gene. In human, some nucleotide mutations of *GALNT1* may cause ovarian cancer [36]. On the other hand, normal *GALNT1* may ensure normal functions of ovary. The characteristic of this gene is still unclear in chickens, and the current study is the first report that its polymorphism is associated with egg quality traits. Another ESW40 associated SNP with genome-wise significance is on GGA3, where there are also three chromosome-wise significant SNPs. These four SNPs are located in a 645 Kb segment that may be a novel QTL as it does not coincide with previously reported QTL or candidate gene for ESW [37], [38], [39]. In this putative QTL region, there are lots of known genes including genes related to DNA modification, transcription, replication and RNA translation (*NEIL2*, *GATA4*, *MCM3* and *TRAM2*); genes related to immune system(*IL17*, *Antimicrobial peptide CHP1* and beta-defensin gene cluster); a gene plays a role in the calcium homeostasis(*EFHC1*). Functions of most genes mentioned above are not fully understood in chickens and need to be studied further, although they have been studied extensively in human.

Significant SNPs differed over time for egg shell weight, and a SNP on GGA11 was found to be significantly associated with ESW60. Fairfull and Gowe noted high correlations over ages in egg quality traits [40], but Abasht *et al.* found that in selected lines, similar populations as used in the present study, correlations between early and late traits were weak [41]. Therefore, it is suggested that the partial genetic independence exists in some egg quality traits at different ages and identifying QTL that differ over time for the same trait is important. The significant SNP associated with ESW60 also showed suggestive association (chromosome-wise significance) with EST60 (combined *P* = 2.04E-06) and ESS40 (combined *P* = 1.72E-06), and it is located in the intron3 of *ZNF536* gene that encodes a kind of DNA binding protein and functions as a transcriptional repressor [42], [43]. This is the first report that *ZNF536* may affect egg shell weight in chickens.

Lots of QTL regions affecting eggshell thickness have been detected by previous linkage studies and they distribute on GGA1, GGA2, GGA5, and GGA7 [37], [38], [44]. Some candidate genes for eggshell thickness were also identified on GGA2, GGA4, GGA8 and GGA9 [5], [6]. In this study, two association signals were found on GGA1 for EST40, which is located in a hypothetical locus *LOC418918* and the other in a known gene *ENOX1*. The region harboring these two SNPs ranges from 171.22 Mb to 179.35 Mb, which may be a novel QTL for eggshell thickness and about 70 Mb away from the QTL reported by Sasaki *et al.* [38].

Internal quality is becoming another focus of attention in improving egg quality traits, especially as egg consumption is changing from shell eggs toward egg products. Yolk weight is an important indication of internal egg quality. In this study, an association signal at genome-wise significant level was found for YW40, located in *ATM* gene on GGA1. *ATM* can regulate a wide variety of downstream proteins mainly related to genome stabling and cell cycle controlling [45], [46], [47]. This is an important candidate gene, but in chickens its function is still unclear and needs future study.

In order to avoid the extreme conservation induced by Bonferroni correction and increase probabilities of finding potential genetic variant impacting egg production and quality traits, association tests were also performed for markers on each chromosome individually. Some chromosome-wise significant SNPs were found in locations within the previously reported QTL regions. On GGA4, there were four QTL regions affecting egg weight, and their range span 51.6–52.6 Mb, 46.7–46.8 Mb, 70.9–80.3 Mb and 61.5–81.3 Mb [38], [48], [49]. In the present study, three SNPs associated with EW40 chromosome-wise are within a segment ranging from 78.67 Mb to 79.36 Mb, which coincides with the QTL regions reported by Sasaki *et al.* and Tuiskula-Haavisto *et al.* [38], [48]. For FEW, the chromosome-wise significant SNPs are located in a QTL region (38.00 Mb to 38.53 Mb) on GGA1 for egg weight at 29 wk in a red junglefowl (RJF)×White Leghorn (WL) cross reported by Kerje *et al.* [49].

As large differences of genetic background and phenotypic distribution exist between White Leghorn and dwarf layers, data from individual studies of these two populations cannot be analyzed together in a single association test. Otherwise it would induce false positive caused by population stratification. Therefore, we conducted association tests separately and then performed a meta-analysis to increase the statistical power in estimating the true effect signals. Fisher's combined probability method, although proposed decades ago, is a simple but elegant technique for meta-analysis. This method is appropriate to combine the results from several independent tests bearing upon the same overall hypothesis. For the present study, association tests in two different experimental populations were under the same null hypothesis (H0): no SNP associated with the trait. Furthermore, as the basis of the combined probability method should be one-sided test, and in order to avoid the heterogeneity, we rejected SNP markers with opposing direction of effect in separate studies, and combined ones with additive effect in the same direction. Therefore, Fisher's method is particularly well suited as a meta-analysis tool for this study that performed association tests across different experimental populations.

The number of identified SNPs is limited and these loci might not fully describe genetic diversity underlying traits in this study. Furthermore, the genetic mechanisms of quantitative traits might involve complex interactions among genes and between genes and environmental conditions, or epigenetic mechanisms which cannot be captured by additive models. Therefore, increasing density of markers in the genotyping panels and improving genetic models and statistical methods may benefit the detection of causative genetic variability for quantitative traits in domestic animals.

In summary, the current study revealed 8 genome-wise significant and 95 chromosome-wise significant SNPs for egg production and quality traits in White Leghorn and brown-egg dwarf layers by using the high-density SNP array and association analysis based on least squares regression. Some SNPs are located in possible causative genes or within the previously reported QTL region, but most of the significant SNPSs are reported, for the first time, to be associated with egg production and quality traits. To our knowledge, this is the first publication of GWAS on egg production and quality traits in chickens and our findings lay a preliminary foundation for follow-up studies to identify causal mutations by enriching markers within the identified intervals and functional studies on related genes, and subsequently they may be applied in marker-assisted selection program on egg layers.

## Supporting Information

Table S1 **Chromosome-wise significant threshold for each chromosome.**
(DOC)

Table S2 **Chromosome-wise significant trait-SNP combinations.**
(DOC)
