# Supplementary material for: A Genome-Wide SNP Scan Reveals Novel Loci for Egg Production and Quality Traits in White Leghorn and Brown-Egg Dwarf Layers
Source: PLoS One. 2011 Dec 8;6(12):e28600. doi: 10.1371/journal.pone.0028600 (PMC3234275; doi:10.1371/journal.pone.0028600)
Supplement: Table S1 — Chromosome-wise significant threshold for each chromosome. (DOC) [file pone.0028600.s001.doc]

**Table S1.**

| **GGA** | **Number of SNP markers** | **Significant threshold (Bonferroni correction)** |
| --- | --- | --- |
| 1 | 5481 | 9.12E-06 |
| 2 | 3905 | 1.28E-05 |
| 3 | 3122 | 1.60E-05 |
| 4 | 2698 | 1.85E-05 |
| 5 | 1666 | 3.00E-05 |
| 6 | 1383 | 3.62E-05 |
| 7 | 1268 | 3.94E-05 |
| 8 | 1133 | 4.41E-05 |
| 9 | 935 | 5.35E-05 |
| 10 | 940 | 5.32E-05 |
| 11 | 786 | 6.36E-05 |
| 12 | 968 | 5.17E-05 |
| 13 | 952 | 5.25E-05 |
| 14 | 786 | 6.36E-05 |
| 15 | 769 | 6.50E-05 |
| 16 | 18 | 2.78E-03 |
| 17 | 678 | 7.37E-05 |
| 18 | 634 | 7.89E-05 |
| 19 | 606 | 8.25E-05 |
| 20 | 1009 | 4.96E-05 |
| 21 | 562 | 8.90E-05 |
| 22 | 181 | 2.76E-04 |
| 23 | 457 | 1.09E-04 |
| 24 | 592 | 8.45E-05 |
| 25 | 113 | 4.42E-04 |
| 26 | 537 | 9.31E-05 |
| 27 | 379 | 1.32E-04 |
| 28 | 435 | 1.15E-04 |
| E22 | 75 | 6.67E-04 |
