# Supplementary material for: A Genome-Wide SNP Scan Reveals Novel Loci for Egg Production and Quality Traits in White Leghorn and Brown-Egg Dwarf Layers
Source: PLoS One. 2011 Dec 8;6(12):e28600. doi: 10.1371/journal.pone.0028600 (PMC3234275; doi:10.1371/journal.pone.0028600)
Supplement: Table S2 — Chromosome-wise significant trait-SNP combinations. (DOC) [file pone.0028600.s002.doc]

**Table S2.**

| **GGA** | **Position(bp)** | **SNP** | **Combined *P* value** | **Associated trait** |
| --- | --- | --- | --- | --- |
| 1 | 13971074 | rs10725499 | 5.38E-06 | AH40 |
| 1 | 38300287 | rs13851731* | 5.10E-06 | FEW |
| 1 | 190154785 | rs13990201 | 5.57E-06 | EST40 |
| 1 | 199125539 | GGaluGA063871 | 7.83E-06 | EST40 |
| 2 | 9145291 | rs14136534 | 4.16E-06 | AFE |
| 2 | 10051436 | GGaluGA133279 | 8.63E-06 | AH40 |
| 2 | 86157489 | GGaluGA155836 | 1.28E-05 | ESW40 |
| 2 | 86166026 | rs16051157 | 2.34E-06 | ESW40 |
| 2 | 122002947 | rs14240104 | 2.86E-06 | EST40 |
| 2 | 122109877 | rs14240170 | 6.47E-06 | EST40 |
| 3 | 42431299 | rs14344183 | 1.02E-05 | EN |
| 3 | 108730042 | rs14409754 | 1.24E-05 | AFE |
| 3 | 110204493 | GGaluGA239405 | 1.84E-06 | ESW40 |
| 3 | 110276487 | GGaluGA239445 | 6.13E-06 | ESW40 |
| 3 | 110740773 | rs14412242 | 1.14E-05 | ESW40 |
| 4 | 72545086 | GGaluGA265049 | 3.15E-06 | AFE |
| 4 | 72570622 | rs16433282 | 3.00E-06 | AFE |
| 4 | 78673906 | rs15619223* | 7.57E-06 | EW40 |
| 4 | 78755454 | rs14491017* | 6.45E-06 | EW40 |
| 4 | 79361533 | GGaluGA266150* | 5.33E-06 | EW40 |
| 5 | 37098969 | GGaluGA282762 | 1.51E-05 | EW40 |
| 5 | 50524623 | rs13590049 | 4.38E-06 | ESW40 |
| 5 | 53885542 | GGaluGA288650 | 1.19E-05 | EW40, EW60 |
| 5 | 54730483 | GGaluGA289107* | 1.10E-05 | EST40 |
| 5 | 54676176 | rs16512242 | 1.02E-05 | EW60 |
| 6 | 3573064 | rs14560805 | 1.33E-05 | EW40 |
| 6 | 4603164 | rs14562446 | 1.07E-05 | ESS60 |
| 6 | 5081654 | rs13562188 | 2.29E-05 | ESS40 |
| 6 | 18845107 | GGaluGA300183 | 2.58E-05 | AFE |
| 6 | 28715622 | rs14588411 | 1.65E-05 | YW40 |
| 7 | 3844740 | rs13739847 | 1.14E-05 | AFE |
| 7 | 11728866 | rs14606249 | 1.20E-05 | EW40 |
| 7 | 15112177 | rs16587332 | 3.49E-05 | HU40 |
| 7 | 21999861 | GGaluGA315104 | 2.03E-05 | EN |
| 7 | 27472791 | rs14619627 | 1.90E-05 | HU40 |
| 7 | 27565899 | rs14619719 | 1.91E-05 | EW40 |
| 7 | 29066504 | GGaluGA318038 | 3.41E-05 | EST40 |
| 8 | 20019332 | rs15922613 | 3.38E-05 | EW60 |
| 8 | 25233276 | GGaluGA330945 | 1.24E-05 | AH60 |
| 9 | 4700548 | rs14670090 | 4.62E-06 | AH60 |
| 10 | 17243856 | rs14010945 | 3.67E-05 | EW40 |
| 10 | 17617664 | rs13785423 | 7.16E-06 | EW40 |
| 11 | 1549113 | GGaluGA074444 | 2.53E-06 | EST40 |
| 11 | 7911259 | rs15608480 | 9.12E-06 | ESW40 |
| 11 | 8975011 | rs14022292 | 2.66E-05 | ESW40 |
| 11 | 9596922 | rs14022717 | 1.72E-06 | EST60, ESS40 |
| 11 | 14669826 | rs14965446 | 1.31E-05 | AFE |
| 11 | 15169257 | GGaluGA078178 | 4.20E-06 | EW40 |
| 11 | 17963253 | rs14027438 | 5.61E-06 | YW40 |
| 11 | 18133483 | rs14966714 | 5.17E-06 | YW40 |
| 12 | 16204627 | rs14045602 | 4.78E-05 | AFE |
| 12 | 19871135 | GGaluGA089865 | 9.29E-06 | EST40 |
| 13 | 4809532 | rs14990366 | 7.77E-06 | AFE |
| 13 | 9007953 | rs16083096 | 1.73E-05 | AH40 |
| 13 | 13030608 | rs14061598 | 8.39E-06 | AFE |
| 14 | 5825512 | rs14074129 | 6.33E | EW40 |
| 14 | 7180811 | GGaluGA102453 | 4.04E | EST40 |
| 14 | 12882155 | GGaluGA104320 | 4.55E | AFE |
| 14 | 13471792 | rs15012784 | 3.54E | HU40 |
| 14 | 14040788 | GGaluGA104992 | 5.87E | ESW40 |
| 15 | 1148750 | GGaluGA106238 | 3.40E | EN |
| 15 | 2141272 | Gga_rs15764239 | 4.68E | EW40 |
| 15 | 2705782 | GGaluGA106954 | 6.38E | EN |
| 15 | 6177532 | Gga_rs15021186 | 1.41E | YW60 |
| 17 | 5095923 | Gga_rs15031855 | 1.70E-06 | EW60 |
| 17 | 5409995 | Gga_rs15799031 | 3.64E-06 | EW60 |
| 17 | 5824883 | GGaluGA114853 | 7.59E-05 | EN |
| 17 | 8606235 | GGaluGA116683 | 1.44E-05 | ESS40 |
| 17 | 8606235 | GGaluGA116683 | 5.12E-05 | EST40 |
| 19 | 4327863 | Gga_rs15047274 | 1.29E-05 | ESS60 |
| 19 | 5828258 | GGaluGA127202 | 4.41E-05 | AFE |
| 20 | 8778348 | Gga_rs16170107 | 2.65E-05 | EW60 |
| 20 | 11613151 | Gga_rs13635060 | 1.90E-05 | HU60 |
| 20 | 13032938 | Gga_rs15179742 | 4.4E-05 | EN |
| 21 | 2090706 | GGaluGA182905 | 1.52E-05 | AFE |
| 21 | 2135314 | Gga_rs16178648 | 6.05E-05 | AFE |
| 21 | 3516345 | Gga_rs14284021 | 1.44E-05 | EST60 |
| 22 | 2962620 | GGaluGA186440 | 1.76E-06 | EN |
| 22 | 3072633 | Gga_rs15190548 | 2.61E-05 | EN |
| 22 | 3289975 | Gga_rs16184839 | 9.15E-06 | EN |
| 22 | 3344637 | Gga_rs13820412 | 5.17E-06 | YW40 |
| 23 | 559003 | Gga_rs16186114 | 1.17E-05 | AH60 |
| 23 | 4305822 | Gga_rs15203613 | 3.96E-05 | EN |
| 24 | 3395170 | Gga_rs16196468 | 8.01E-05 | EST40 |
| 24 | 3942494 | GGaluGA192546 | 4.87E-05 | AH60 |
| 26 | 1814654 | Gga_rs15229556 | 2.95E-06 | YW40 |
| 26 | 1986427 | GGaluGA196081 | 1.62E-05 | EW40 |
| 26 | 2255685 | GGaluGA196269 | 2.73E-05 | AFE |
| 26 | 3315110 | GGaluGA197072 | 3.67E-06 | FEW |
| 26 | 4169386 | Gga_rs13606694 | 8.12E-05 | ESS60 |
| 27 | 4079776 | Gga_rs10723293 | 5.17E-05 | EW40 |
| 27 | 4601339 | Gga_rs16208202 | 5.11E-05 | EST40 |
| 28 | 840056 | GGaluGA201096 | 7.01E-05 | ESS60 |
| 28 | 2574133 | GGaluGA202286 | 7.34E-05 | HU40 |
| 28 | 2934188 | Gga_rs15251169 | 0.000109 | HU40 |

*SNPs located in the previously reported QTL regions
